# Supplementary material for: Striated muscle-specific base editing enables correction of mutations causing dilated cardiomyopathy
Source: Nat Commun. 2023 Jun 22;14:3714. doi: 10.1038/s41467-023-39352-1 (PMC10287752; doi:10.1038/s41467-023-39352-1)
Supplement: Supplementary file 1 — Supplementary Information [file 41467_2023_39352_MOESM1_ESM.pdf]

Supplementary Figures

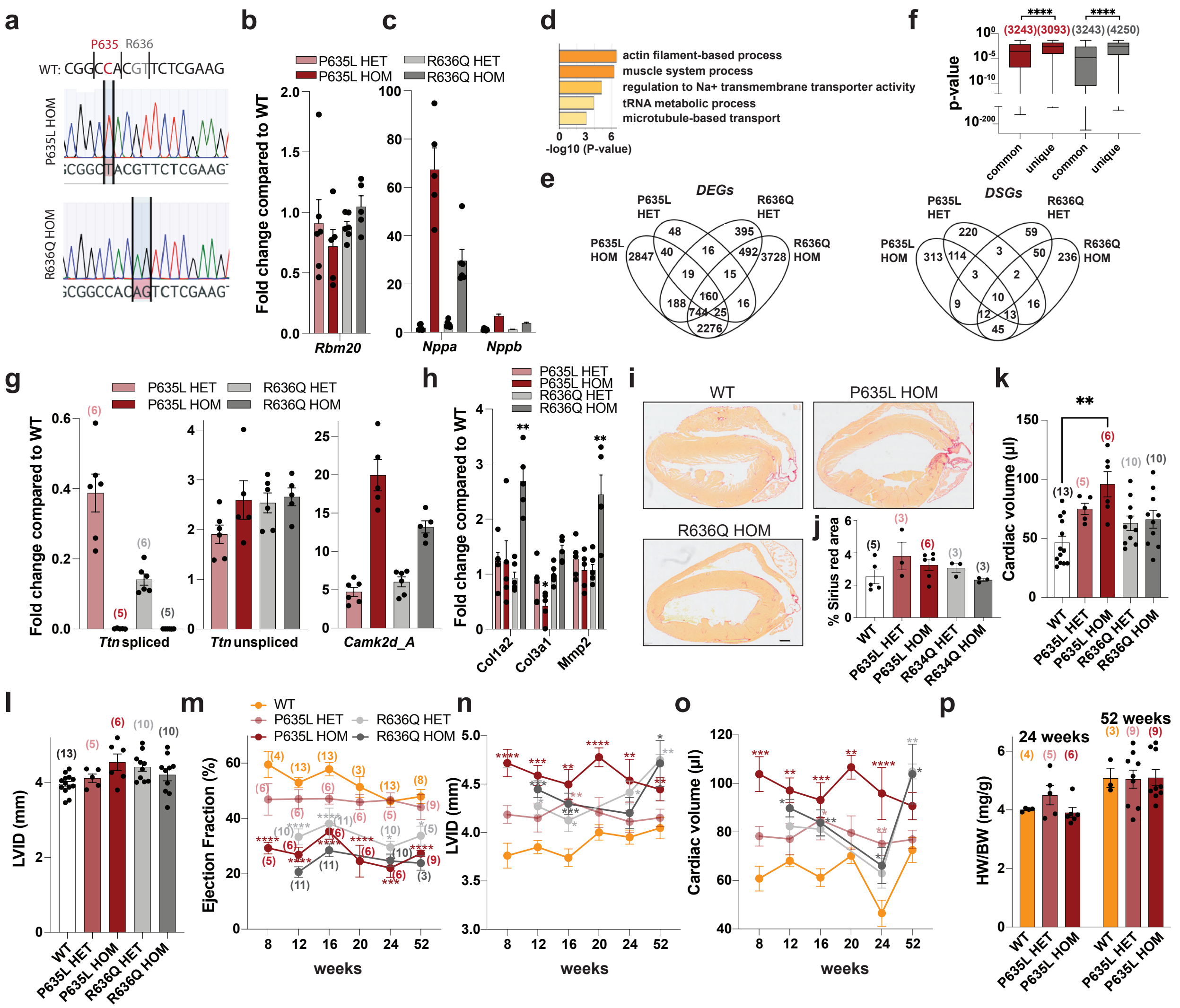

Supplementary Figure 1: Extended characterization of P635L and R636Q mouse lines. a) Sanger sequencing traces of first generation homozygous mutant mice used for subsequent mating and experiments. Red (P635L): C>T mutation; grey (R636Q): GT>AG mutation. Note that for subsequent base editing in R636Q, the CAG codon is converted to CGG which is synonymous to the WT CGT codon. **b, c)** Expression fold change compared to WT of *Rbm20* (**b**), and *Nppa* and *Nppb* (**c**). mice. **d)** GO analysis (biological function) of DSGs that overlap for both P635L and R636Q HOM mice with a cut-off of p_{adjust} <0.01 and ΔPSI > 0.1. **e)** Venn diagram of significantly DEGs (up, p_{adjust} <0.05) and DSGs (down, p_{adjust} <0.01 and ΔPSI > 0.1). **f)** P-values (cut-off p_{adjust} <0.05) of DEGs unique or common between P635L (red) and R636Q HOM (grey). Significant changes analyzed by unpaired, two-tailed t-tests. **** p< 0.0001. Boxplots depict the median with the box including the 25-75th percentile and the whiskers ranging from the smallest to the largest value. The number of genes is shown above the plots. **g, h)** Expression fold change compared to WT of spliced and unspliced *Ttn* isoforms and *Camk2d* isoform A (**g**) or fibrosis marker genes (**h**)

determined by qPCR. Significant changes indicated and analyzed by unpaired, two-tailed t-tests. * $p < 0.05$, ** $p < 0.01$. **i, j)** Representative heart tissue sections stained with Sirius Red (**i**) and quantification of Sirius Red positive area (**j**). Scale bar: 500 μm. **k, l)** Cardiac volume (**k**) and LVID (**l**) determined by narcosis echocardiography. Only significant differences are labelled. P-values obtained from one-way ANOVA with Tukey`s multiple comparison test: ** $p < 0.01$. Mice were 24-weeks old. **m-o)** Percentage of ejection fraction (**m**), LVID (**n**) and cardiac volume (**o**) determined by time-course narcosis echocardiography of *Rbm20* mutant mice. Asterisk indicates statistical significance compared to WT obtained by two-way ANOVA with Tukey`s multiple comparison test: **** $p < 0.0001$, *** $p < 0.001$, ** $p < 0.01$, * $p < 0.05$. Data for the 24 week time point is the same as in Fig. 1j and Sup. Fig. 1j, k. Data for R636Q HET and HOM at 12 and 16 weeks has been published by us before[1]. (**p**) Heart-to-body weight ratio after 24 and 52 weeks of *P635L* mutant mice. N=5 for HOM and 6 for HET mice unless indicated otherwise in brackets above the bars. No significant changes were found in (**j**), (**l**) and (**p**). All data was obtained in 16-week-old mice if not indicated otherwise. Error bars depict the SEM in all panels. Gene expression analysis was performed using RNA isolated from left ventricles.

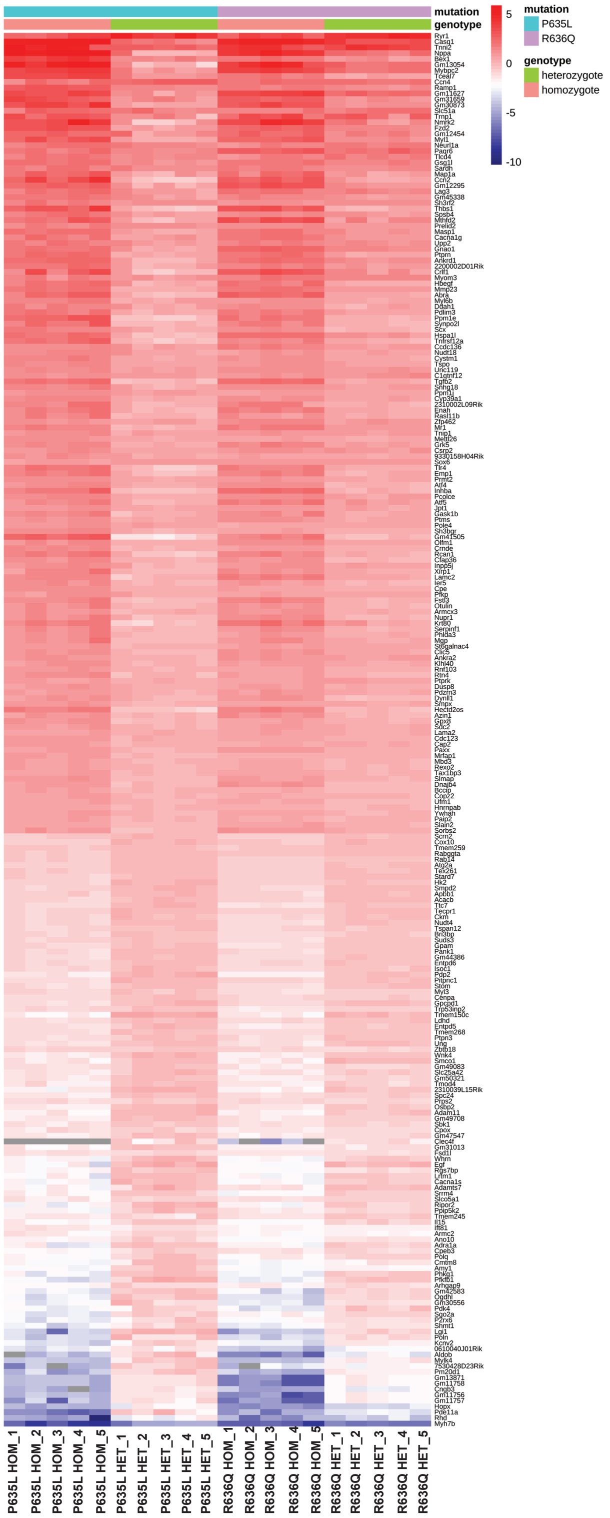

Supplementary Figure 2: Heatmap of DEGs of P635L and R636Q mice derived from RNA-seq. Grey bars indicate that the gene was not detected in RNA-seq data. N = 5 mice per genotype. All DEGs that overlap in P635L and R636Q HOM mice are shown with a stringent cut-off of p_{adjust} <1e^{-10}.

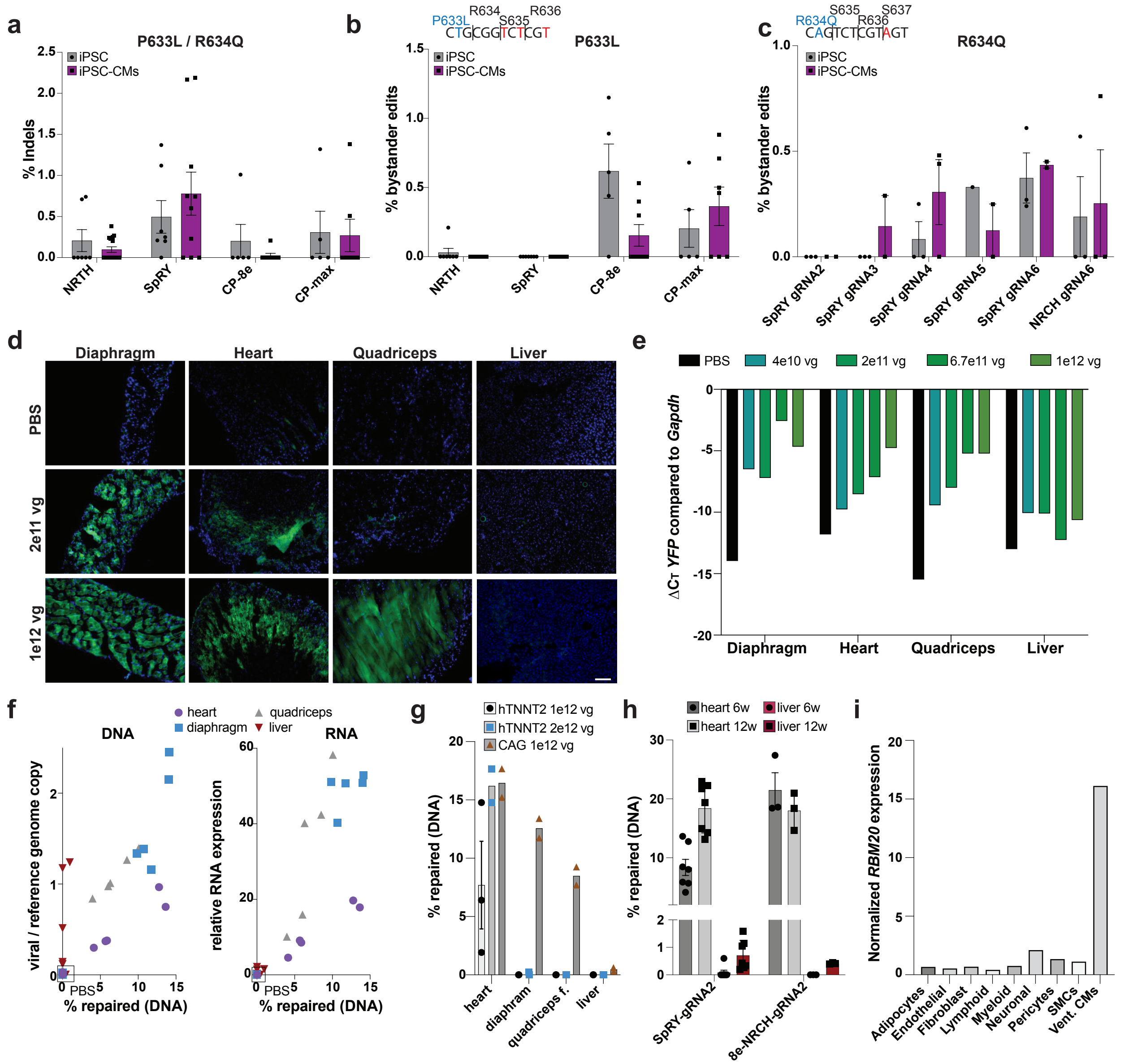

Supplementary Figure 3: Analysis of base editing in iPSC-CMs and in mice. a-c) Percentage of Indels (**a**) and bystander edits in P633L (**b**) and R634Q (**c**) iPSCs and iPSC-CMs. Indel formation is the summed frequency of insertions or deletions in a window of 10 bp upstream to 10 bp downstream of the gRNA binding site. Indel formation is shown combined for both mutations and gRNAs and separated by different base editors only. Bystander edits are sequences that contain extra A>G conversions within the gRNA window. Observed positions of bystander edits is indicated in red, the on-target site is in blue. **d, e)** Representative tissue sections (**d**) and RNA expression data (**e**) measuring the fluorescent transgene *YFP* delivered by AAVMYO and injected in different concentrations in WT mice. Scale bar: 100 µm. One mouse per concentration was injected. **f)** Percentage of repaired reads relative to viral copy number per diploid genome (**left**) or relative to RNA expression (**right**) determined by ddPCR in the muscle tissues heart, diaphragm and quadriceps femoris (quadriceps f.), as well as the liver. For the left panel, DNA was used as input with primers for the CMV promoter, for the right panel, RNA reverse transcribed

to cDNA was used with primers for the transcribed WPRE element common in all base editor constructs. Only the SpRY-gRNA2 combination was analyzed. Each datapoint represents one mouse. **g)** Editing efficacy of NRCH / gRNA2 driven by the *hTNNT2* or the *CAG* promoter. Concentration shown is the combined amount of N-and C-terminal base editor-containing AAV. N = 2 mice (CAG 1e12 and hTNNT2 2e12, no error bars) or 3 mice (hTNNT2 1e12). **h)** Editing efficacy of SpRY and 8e-NRCH in heart and liver 6 and 12 weeks after injection in 4-week-old mice. N= 3 (8e-NRCH-gRNA2) or 7 (SpRY-gRNA2) mice. **i)** Normalized RNA expression of *RBM20* derived from single-nucleus RNA-seq of the human heart[2]. SMCs = smooth muscle cells, Vent. CMs = ventricular cardiomyocytes. Error bars depict the SEM in all panels. Only P635L HOM were treated.

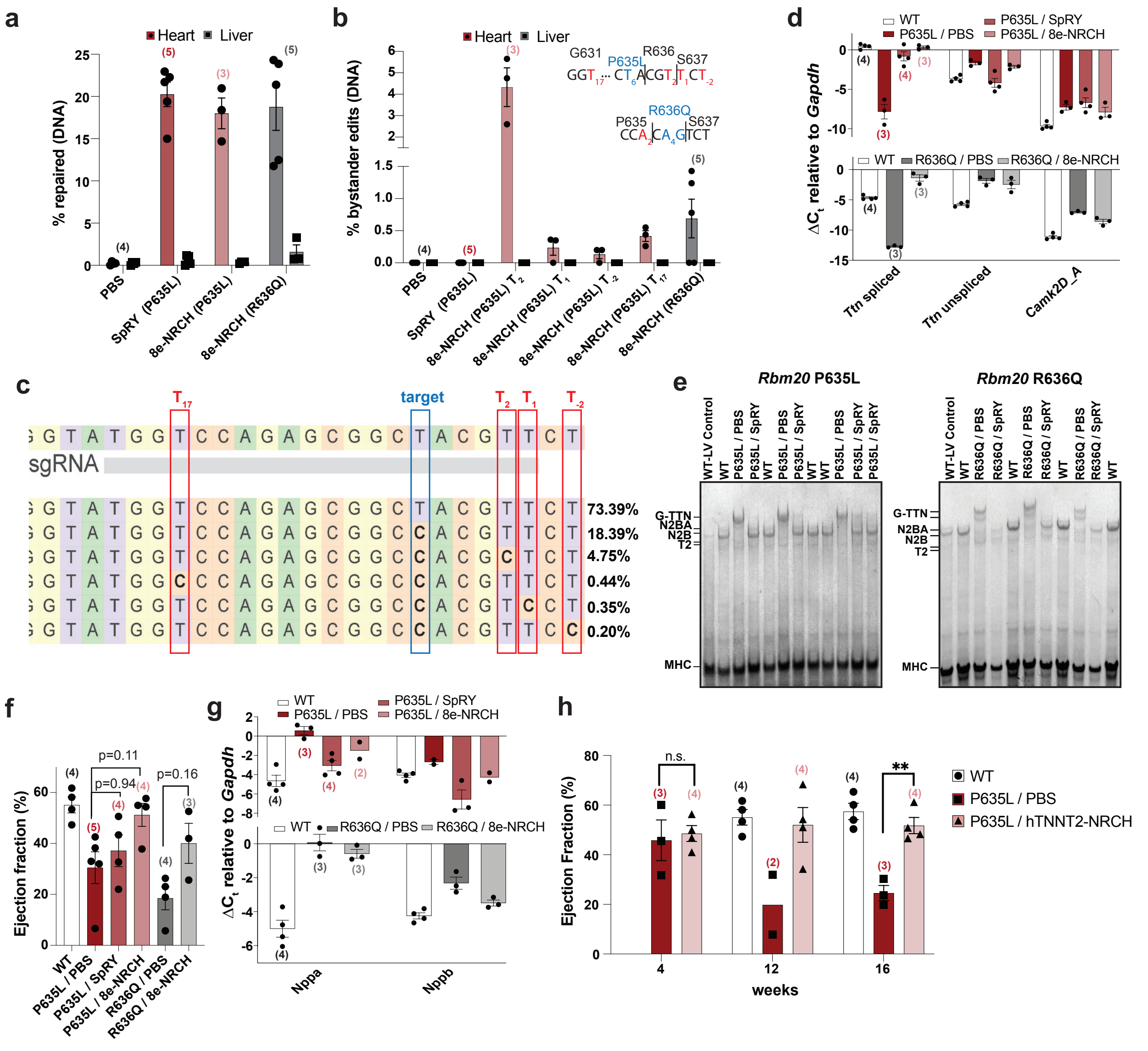

Supplementary Figure 4: Extended phenotypic characterization of mice after AAVMYO-ABE treatment. a, b) Allele frequency of repaired DNA (**a**) and bystander edits (**b**) in mice treated with AAVMYO-ABE. Sequence shows location of the on-target edit in blue and the bystander edits in red. Numbers depict the position of the nucleotides within the targeting gRNA with the PAM sequence in position 21-23. **c)** Example allele frequency determined by Crispresso2 for one mouse treated with AAVMYO harboring the base editor 8e-NRCH and gRNA2. On-target location is indicated in blue and location of observed bystander edits in red. **d)** Expression of spliced and unspliced *Ttn* isoforms and *Camk2d* isoform A in WT and mutant mice treated with PBS or AAVMYO-ABE. **e)** Vertical TTN agarose gels showing the G-N2BA, N2BA N2B, and T2 protein isoforms of TTN, and MHC as a loading control**. f)** Percentage of ejection fraction in WT or mutant mice 8 weeks after injection with PBS or AAVMYO-ABE. P-values obtained by one-way ANOVA with Tukey`s multiple comparison test shown for AAVMYO versus PBS. N = 3-5 mice per condition. **g)** Expression of heart failure biomarkers *Nppa* and *Nppb* in WT and mutant mice treated with PBS or AAVMYO-ABE. **h)** Ejection fraction before (at week 4) and 8 and 12 weeks

after injection of AAVMYO-ABE with hTNNT2-driven NRCH base editor. Twice the amount of AAVMYO-ABE was used compared to CAG-driven ABEs. In contrast to other experiments, we performed echocardiography also before the injection showing that PBS and ABE-treated mice had similar physiological parameters. WT data was obtained separately and is the same as in Fig. 3h. Statistical significance was assessed by unpaired, two-tailed t-tests between PBS and ABE-treated mice. ** $p < 0.01$, n.s. = not significant. Only P635L or R636Q HOM mice were treated. Error bars depict the SEM in all panels. The number of mice per condition is indicated in the graphs. All data except in (**e**) and (**g**) were obtained 12 weeks after AAVMYO-ABE injection. DNA, RNA and protein isolated from left ventricles.

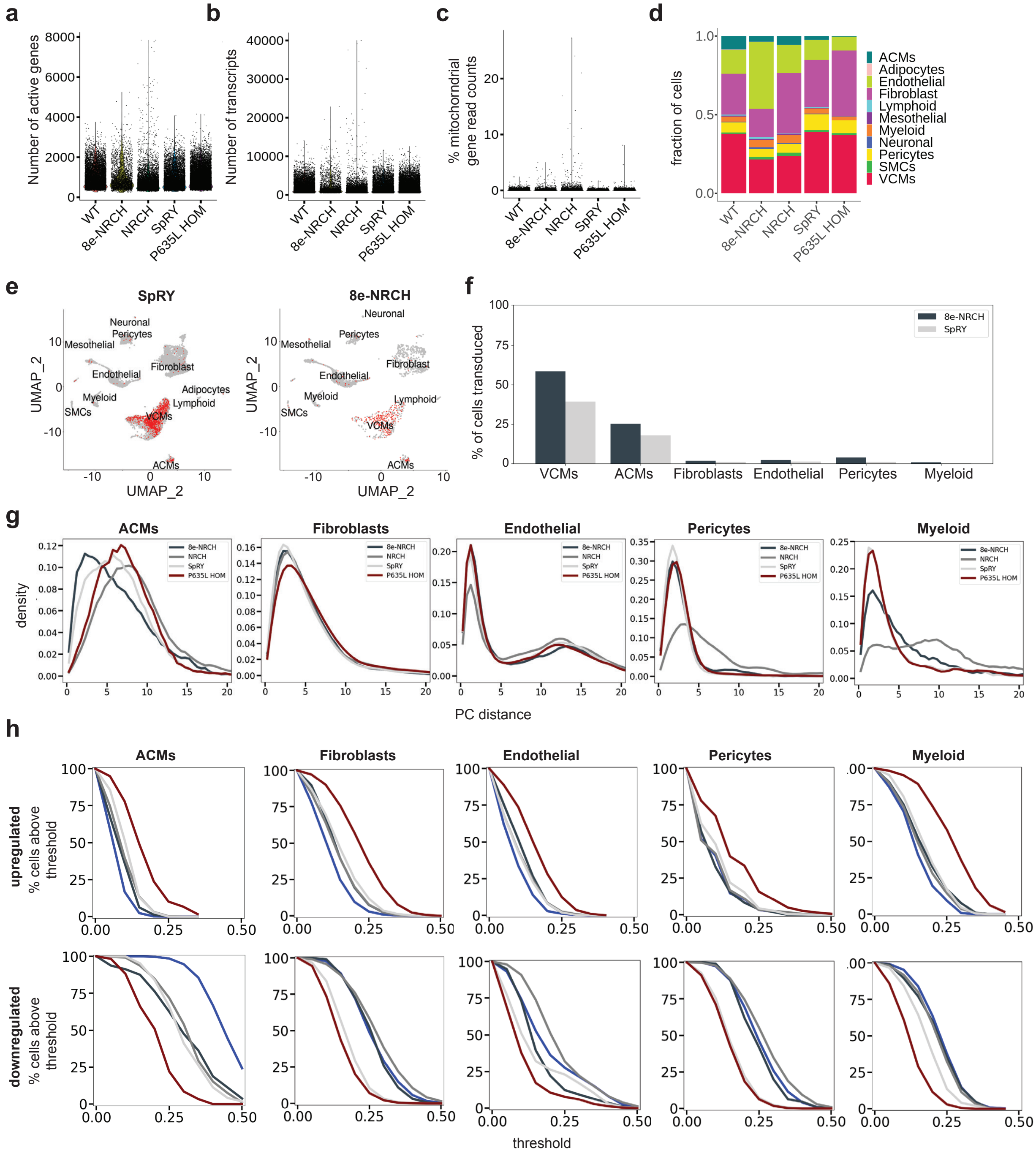

Supplementary Figure 5: Extended snRNA-seq analysis. a-c) Number of active genes (**a**), total transcript counts (**b**) and percentage of mitochondrial gene counts per cell (**c**) for nuclei from each condition. Two independent snRNA-seq experiments were performed for each condition. **d)** Relative cell type distribution in WT, P635L HOM and base edited mice. **e, f)** UMAPs (**e**) and quantification of the fraction of cells expressing the base editor construct (**f**) delivered by AAVMYO. N-and C-terminal base editor expression values were summed up. Values are either 0 (not expressed, grey) or 1 (expressed, red). **g)** Histograms depicting the distribution of pairwise Euclidean distances of depicted cell types from P635L HOM and base edited mice relative to WT

upon mapping using two principal components (PC). **h)** Threshold of activity score of depicted cell types (see methods for calculation) relative to percentage of cells above the threshold for genes upregulated (upper panel) or downregulated (lower panel) in P635L HOM relative to WT.

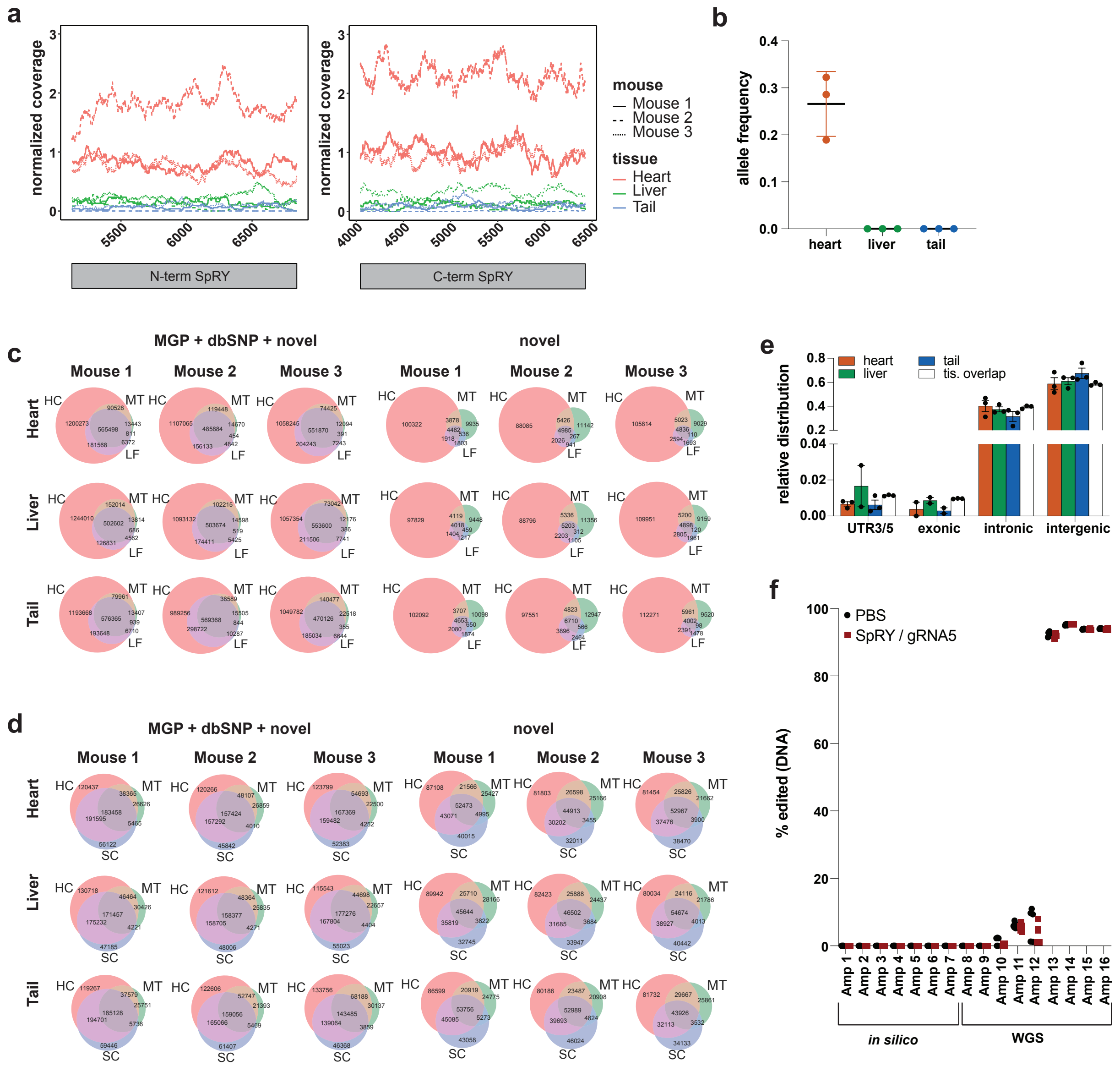

Supplementary Figure 6: AAV coverage and editing events detected by WGS. a) Normalized read coverage across autosomes of C- and N-terminal base editor sequence delivered by AAVMYO. **b)** Allele frequency of the P635L A>G nucleotide conversion. **c, d)** All (left) and novel (right) SNVs (**c**) or Indels (**d**) called by four variant callers (HC: HaplotypeCaller, MT: Mutect2, LF: Lofreq, SC: Scalpel). **e)** Mean relative distribution of tissue-specific and common variants within coding or non-coding regions of the genome. Tissue overlap represent variants that were common to all three tissues. **f)** Allele frequency of 16 candidate loci analyzed by amplicon-seq. Seven loci were determined by *in silico* predictions of off-target editing based on gRNA sequence similarity. Nine loci were obtained from WGS by taking A>G / T>C SNVs with the coverage. N = 5 mice treated with PBS and 5 mice treated with AAVMYO-SpRY. Error bars depict the SEM in all panels.

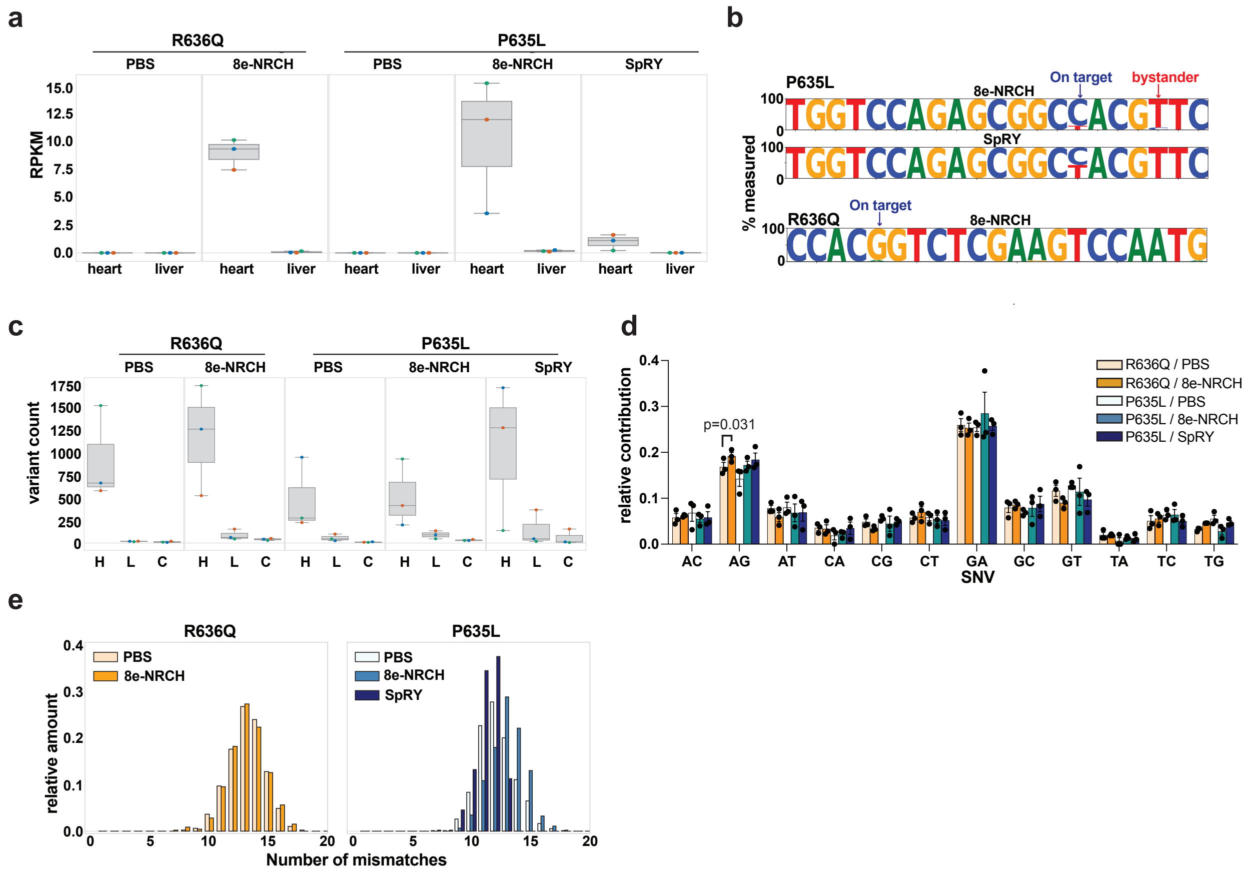

Supplementary Figure 7: RNA on- and off-target editing. a) Base editor expression in heart and liver tissue 12 weeks after AAVMYO-ABE or PBS treatment in P635L or P636Q HOM mice. RPKM = reads per kilobase million. **b)** On-target edit (blue arrow) and bystander edit (red arrow) within the gRNA region targeting *Rbm20* on chromosome 19. Shown is the percentage of each base aligned to the positions plotted on the x-axis for R636Q and P635L HOM mice. In each row, three replicated were summed before calculating the fractions. **c)** Number of heart (H), liver (L) and common variants after filtering as described in the method section. **d)** Averaged relative amount of distinct types of SNVs identified as heart-specific. Significance tests were performed with a logistic regression model using the python package statsmodels and testing the difference in A<G vs. non-A<G ratios between pairs of ABE and PBS-treated samples. Error bars depict standard deviation. **e)** Number of mismatches to the gRNA and PAM sequence in the area of ± 30 bases around the variant start site. Variants from three replicates were summed up. Variants on the X or Y chromosome were excluded. Error bars depict the SEM in all panels. Box plots in (**a**) and (**c**) depict the median including the 25th-75th percentile with whisikers extending to the rest of the distribution.

References:

1. Lennermann, D. C. *et al.* Deep phenotyping of two preclinical mouse models and a cohort of RBM20 mutation carriers reveals no sex-dependent disease severity in RBM20 cardiomyopathy. *Am J Physiol Heart Circ Physiol* **323**, H1296–H1310 (2022).
2. Litviňuková, M. *et al.* Cells of the adult human heart. *Nature* **588**, 466–472 (2020).
