# Supplementary material for: Striated muscle-specific base editing enables correction of mutations causing dilated cardiomyopathy
Source: Nat Commun. 2023 Jun 22;14:3714. doi: 10.1038/s41467-023-39352-1 (PMC10287752; doi:10.1038/s41467-023-39352-1)
Supplement: Supplementary file 2 — Reporting Summary [file 41467_2023_39352_MOESM2_ESM.pdf]

nature portfolio

Corresponding author(s): Lars Steinmetz

Last updated by author(s): May 31, 2023

Reporting Summary

Nature Portfolio wishes to improve the reproducibility of the work that we publish. This form provides structure for consistency and transparency in reporting. For further information on Nature Portfolio policies, see our Editorial Policies and the Editorial Policy Checklist.

Statistics

For all statistical analyses, confirm that the following items are present in the figure legend, table legend, main text, or Methods section.

n/a	Confirmed	
☐	☒	The exact sample size (*n*) for each experimental group/condition, given as a discrete number and unit of measurement
☐	☒	A statement on whether measurements were taken from distinct samples or whether the same sample was measured repeatedly
☐	☒	The statistical test(s) used AND whether they are one- or two-sided<br>*Only common tests should be described solely by name; describe more complex techniques in the Methods section.*
☒	☐	A description of all covariates tested
☒	☐	A description of any assumptions or corrections, such as tests of normality and adjustment for multiple comparisons
☐	☒	A full description of the statistical parameters including central tendency (e.g. means) or other basic estimates (e.g. regression coefficient) AND variation (e.g. standard deviation) or associated estimates of uncertainty (e.g. confidence intervals)
☐	☒	For null hypothesis testing, the test statistic (e.g. *F*, *t*, *r*) with confidence intervals, effect sizes, degrees of freedom and *P* value noted<br>*Give P values as exact values whenever suitable.*
☒	☐	For Bayesian analysis, information on the choice of priors and Markov chain Monte Carlo settings
☒	☐	For hierarchical and complex designs, identification of the appropriate level for tests and full reporting of outcomes
☒	☐	Estimates of effect sizes (e.g. Cohen's *d*, Pearson's *r*), indicating how they were calculated

Our web collection on statistics for biologists contains articles on many of the points above.

Software and code

Policy information about availability of computer code

Data collection	No software was used.
Data analysis	Data was analyzed with GraphPad Prism software (v9.3.1) and R (v4.2.2). DEseq2 (v3.17) and rMATS (v4.1.2) were used for the analysis of bulk RNA-seq. RNA variants were called with GATK HaplotypeCaller v.4.1.9.0, Strelka v.2.9.10 and Platypus v.0.8.1 and reads were processed with Opossum v.0.2. Metascape (v3.5.20230501) was used for Gene Ontology analysis. For the WGS analysis, we used cutadapt (v.3.5), bwa-mem (v.0.7.17), GATK Mutect2 v4.1.9.0, GATK HaplotypeCaller v.4.1.9.0 , Lofreq v.2.1.5, Scalpel v.0.5.4 and bcftools (v.1.9. SnRNA-Seq data was aligned to the mouse reference mm10 (GENCODE vM23/Ensembl 98) using 10x Cell Ranger 7.0. Downstream analysis on the gene count matrix was performed in R v4.2.1 and Seurat v4. Details are provided in the method section.

For manuscripts utilizing custom algorithms or software that are central to the research but not yet described in published literature, software must be made available to editors and reviewers. We strongly encourage code deposition in a community repository (e.g. GitHub). See the Nature Portfolio guidelines for submitting code & software for further information.

Data

Policy information about availability of data

All manuscripts must include a data availability statement. This statement should provide the following information, where applicable:

- Accession codes, unique identifiers, or web links for publicly available datasets
- A description of any restrictions on data availability
- For clinical datasets or third party data, please ensure that the statement adheres to our policy

WGS data was uploaded to the SRA database at NCBI under the accession code SRP415422: https://www.ncbi.nlm.nih.gov/bioproject/917230.
Bulk RNA-seq data was uploaded to NCBI GEO under the accession code GSE226130:
https://www.ncbi.nlm.nih.gov/geo/query/acc.cgi?acc=GSE226130.
Single nuclei RNA-seq data was uploaded to NCBI GEO under the accession code PRJNA960908: https://www.ncbi.nlm.nih.gov/bioproject/PRJNA960908.
WGS and SnRNA-Seq data was aligned to the mouse reference mm10 (GENCODE vM23/Ensembl 98), for bulk RNA-seq analysis, the GENCODE mouse annotation version vM29 with the primary assembly GRCm39 genome was used.
Supplementary tables 2, 3, 4 and 6 containing the list of differentially expressed (bulk and single-nuclei) and spliced genes, as well as the list of tissue-specific variants has been made public on Figshare.
https://figshare.com/projects/Striated_muscle-specific_base_editing_enables_correction_of_mutations_causing_dilated_cardiomyopathy/156347

Human research participants

Policy information about studies involving human research participants and Sex and Gender in Research.

Reporting on sex and gender	N/A
Population characteristics	N/A
Recruitment	N/A
Ethics oversight	N/A

Note that full information on the approval of the study protocol must also be provided in the manuscript.

Field-specific reporting

Please select the one below that is the best fit for your research. If you are not sure, read the appropriate sections before making your selection.

☒ Life sciences ☐ Behavioural & social sciences ☐ Ecological, evolutionary & environmental sciences

For a reference copy of the document with all sections, see nature.com/documents/nr-reporting-summary-flat.pdf

Life sciences study design

All studies must disclose on these points even when the disclosure is negative.

Sample size	No sample size calculation was performed. Experiments were performed mostly in at least three biological replicates which was sufficient to derive meaningful conclusions. For mouse experiments, mostly five animals were used since this was enough to achieve a statistically measurable effect.
Data exclusions	No data was excluded.
Replication	In vitro cell differentiations were performed independently at least three times. Dependent on the experiment, 3-5 mice were used which were treated as biological replicates. For some experiments, due to the nature of the experiments, we acquired data from more than 5 mice. All attempts for replication were successful except when mice died prematurely or iPSC differentiations did not yield beating cardiomyocytes. In these cases, no data was obtained.
Randomization	All treatments performed in this study were allocated randomly. This includes base editor and control treatment of mice, iPSCs and iPSC-CMs.
Blinding	Blinding was not possible since sample allocation and experiments were performed by the same person.

Reporting for specific materials, systems and methods

We require information from authors about some types of materials, experimental systems and methods used in many studies. Here, indicate whether each material, system or method listed is relevant to your study. If you are not sure if a list item applies to your research, read the appropriate section before selecting a response.

Materials & experimental systems

n/a	Involved in the study
☐	☒ Antibodies
☐	☒ Eukaryotic cell lines
☒	☐ Palaeontology and archaeology
☐	☒ Animals and other organisms
☒	☐ Clinical data
☒	☐ Dual use research of concern

Methods

n/a	Involved in the study
☒	☐ ChIP-seq
☒	☐ Flow cytometry
☒	☐ MRI-based neuroimaging

Antibodies

Antibodies used	anti-Rbm20 (polyclonal, Invitrogen, Catalog # PA5-58068), anti-sarcomeric alpha-actinin (clone EA-53, Abcam, Catalog # ab9465), both diluted 1:250) and anti-rabbit-HRP (12-348, Sigma) were used. The secondary antibodies Alexa Fluor 488 goat anti-mouse IgG (polyclonal, Invitrogen, Catalog #A11001) and Alexa Fluor 568 goat anti-rabbit IgG (polyclonal, Invitrogen, Catalog #A110011,) both diluted 1:1000 were used.
Validation	Anti-Rbm20 antibody was validated by immunohistochemistry by Invitrogen, anti-sarcomeric alpha-actinin was avalidated by immunohistochemistry and western blot by Abcam. Secondary antibodies were validated by immunofluorescence by Invitrogen.

Eukaryotic cell lines

Policy information about cell lines and Sex and Gender in Research

Cell line source(s)	iPSCs generated in Briganti etl al (PMID: 32905764) were used. For AAVMYO production, AAV-293 cells (Stratagene/Agilent) were used. For AAV9 production, HEK293/17 cells (ATCC; CRL-11268) were used.
Authentication	None of the cell lines were authenticated.
Mycoplasma contamination	Cells were negative for mycoplasma before the start of the experiments.
Commonly misidentified lines (See ICLAC register)	No commonly misidentified cell lines were used in this study.

Animals and other research organisms

Policy information about studies involving animals; ARRIVE guidelines recommended for reporting animal research, and Sex and Gender in Research

Laboratory animals	This study was mainly performed in the hybrid mouse strain B6C3F1 backcrossed to C57BL/6J. The animals were maintained in individually ventilated plastic cages (Tecniplast) in an air-conditioned (temperature 22 ± 2 ºC, humidity 50 ± 10%) and light-controlled room (illuminated from 07:00 to 19:00). Mice were fed 1318 P autoclavable diet (Altromin, Germany) ad libitum. Most experiments were performed in at least three mice per condition expect for the eYFP titration experiment where only one mouse per AAV concentration was used (Sup. Fig. 3d, e), the snRNA-seq experiments with only two mice per condition (Fig. 4), and when mice died prematurely before the end of the experiment. The number of mice for all experiments is indicated in the figure legend. For the analysis, the animals were mostly 16 weeks of age except if otherwise indicated in the figure.
Wild animals	No wild animals were used in this study.
Reporting on sex	Mice of both sexes were analyzed and since no overt differences were visible, no discrimination based on sex was performed in the figures. Only for the bulk RNA sequencing, we used only male mice to account for potential confounding effects due to gender differences.
Field-collected samples	No field-collected animals were used in this study.
Ethics oversight	All animal care and procedures performed in this study conformed to the EMBL guidelines for the Use of Animals in Experiments and were reviewed and approved by the Institutional Animal Care and Use Committee (IACUC).

Note that full information on the approval of the study protocol must also be provided in the manuscript.
