# Supplementary material for: Striated muscle-specific base editing enables correction of mutations causing dilated cardiomyopathy
Source: Nat Commun. 2023 Jun 22;14:3714. doi: 10.1038/s41467-023-39352-1 (PMC10287752; doi:10.1038/s41467-023-39352-1)
Supplement: Supplementary file 4 — Description of Additional Supplementary Files [file 41467_2023_39352_MOESM4_ESM.pdf]

Title: Supplementary data 1:
Description: List of significantly differentially expressed genes in P635L HET, P635L HOM, R636Q HET and R636Q HOM relative to WT

Title: Supplementary data 2:
Description: List of significantly differentially spliced events in P635L HET, P635L HOM, R636Q HET and R636Q HOM relative to WT

Title: Supplementary data 3:
Description: List of significantly differentially expressed genes in different cell types based on snRNA-seq including candidate genes for analysis in Fig. 4e-g

Title: Supplementary data 4:
Description: Mapping statistics of NGS experiments including WGS coverage

Title: Supplementary data 5:
Description: Tissue-specific variants found by WGS Supplementary data 6: Sequences of oligonucleotides used in this study
